# The efficiency of retrospective artifact correction methods in improving the statistical power of between-group differences in spinal cord DTI

**DOI:** 10.1016/j.neuroimage.2017.06.051

**Published:** 2017-09

**Authors:** Gergely David, Patrick Freund, Siawoosh Mohammadi

**Affiliations:** a Spinal Cord Injury Center Balgrist, Balgrist University Hospital, Zurich, Switzerland; b Department of Systems Neuroscience, Medical Center Hamburg-Eppendorf, Hamburg, Germany; c Wellcome Trust Centre for Neuroimaging, UCL Institute of Neurology, University College London, London, United Kingdom; d Department of Brain Repair and Rehabilitation, UCL Institute of Neurology, University College London, London, United Kingdom; e Department of Neurophysics, Max Planck Institute for Human Cognitive and Brain Sciences, Leipzig, Germany

## Abstract

Diffusion tensor imaging (DTI) is a promising approach for investigating the white matter microstructure of the spinal cord. However, it suffers from severe susceptibility, physiological, and instrumental artifacts present in the cord. Retrospective correction techniques are popular approaches to reduce these artifacts, because they are widely applicable and do not increase scan time.

In this paper, we present a novel outlier rejection approach (*reliability masking*) which is designed to supplement existing correction approaches by excluding irreversibly corrupted and thus unreliable data points from the DTI index maps. Then, we investigate how chains of retrospective correction techniques including (i) registration, (ii) registration and robust fitting, and (iii) registration, robust fitting, and reliability masking affect the statistical power of a previously reported finding of lower fractional anisotropy values in the posterior column and lateral corticospinal tracts in cervical spondylotic myelopathy (CSM) patients.

While established post-processing steps had small effect on the statistical power of the clinical finding (slice-wise registration: −0.5%, robust fitting: +0.6%), adding reliability masking to the post-processing chain increased it by 4.7%. Interestingly, reliability masking and registration affected the t-score metric differently: while the gain in statistical power due to reliability masking was mainly driven by decreased variability in both groups, registration slightly increased variability. In conclusion, reliability masking is particularly attractive for neuroscience and clinical research studies, as it increases statistical power by reducing group variability and thus provides a cost-efficient alternative to increasing the group size.

## Highlights

• A novel outlier rejection technique (*reliability masking*) is introduced.
• Standard artifact correction has little effect on the statistical power of between-group differences.
• Reliability masking improves the statistical power of between-group differences.
• This improvement is driven by decreased group-level variability.

## 1 Introduction

Diffusion tensor imaging (DTI) is based on the acquisition of diffusion-weighted MR images (Le Bihan and Breton, 1985, Merboldt et al., 1985, Le Bihan et al., 1986) and provides information about the tissue microstructure of the central nervous system. DTI characterizes the magnitude, anisotropy, and orientation of the water diffusion in each voxel using a diffusion tensor model (Basser et al., 1994a, Basser et al., 1994b, Pierpaoli et al., 1996). In contrast to the brain, the white matter (WM) in the spinal cord has a geometry with tightly packed and mostly parallel aligned bundles of axons in rostral-caudal direction, where the DTI signal is less influenced by the sparsely appearing crossing fibers. As a consequence, DTI indices in the spinal cord can be more readily associated with the spinal cord microstructure. For example, radial diffusivity in the spinal cord has been shown to most closely correlate with myelin content, while fractional anisotropy and axial diffusivity have proved to be more indicative of axonal integrity and axonal degeneration (Budde et al., 2007, Budde et al., 2008, Zhang, 2010, Brennan et al., 2013). Furthermore, spinal cord DTI has been successfully related to various disorders with spinal cord involvement (Cohen-Adad et al., 2011, Freund et al., 2012, Grabher et al., 2016, Wheeler-Kingshott et al., 2013).

However, spinal cord DTI is technically challenging and considerably lags behind brain DTI in terms of standardization. Challenges specific for spinal cord DTI include susceptibility artifacts and physiological noise (e.g. due to cardiac pulsation, respiratory motion, and cerebro-spinal fluid (CSF) flow) (Barker, 2001, Stroman et al., 2014). Furthermore, its unfavorable position in the body and the high axial resolution necessary to robustly delineate gray and white matter can lead to severe instrumental artifacts including eddy currents (Jezzard et al., 1998, Mohammadi et al., 2010), vibration artifacts (Gallichan et al., 2010, Mohammadi et al., 2012a), gradient inhomogeneities (Bammer et al., 2003, Nagy et al., 2007, Mohammadi et al., 2012b), and transmit RF field inhomogeneities (Lutti et al., 2012).

To reduce the aforementioned artifacts in spinal cord DTI, optimized acquisition strategies including cardiac-gated (Wheeler-Kingshott et al., 2002a, Wheeler-Kingshott et al., 2002b, Cohen-Adad et al., 2011) and reduced field-of-view sequences (Wheeler-Kingshott et al., 2002a, Wheeler-Kingshott et al., 2002b, Finsterbusch, 2009, Finsterbusch, 2012, Rossi et al., 2008) have been combined with retrospective correction methods. The most commonly applied retrospective correction methods include (i) registration-based methods to reduce misalignment (caused by e.g. subject motion) and image distortions (caused by e.g. eddy currents) (Xu et al., 2013, Mohammadi et al., 2013, Middleton et al., 2014), and (ii) robust tensor fitting techniques (e.g. RESTORE (Chang et al., 2005), PATCH (Zwiers, 2010), and ACID robust tensor fitting (Mohammadi et al., 2013)), to remove the effect of signal outliers (caused by e.g. subject motion or cardiac pulsation) by discarding or down-weighting them in the tensor fit.

While several studies have demonstrated the potential of these retrospective correction methods to improve data quality and yield more reliable tensor estimates at single-subject level, their effect on group differences is still understudied. Investigating the effect of retrospective correction on between-group differences is particularly relevant for various reasons: (i) data acquired at clinical or neuroscience sites might have lower quality and higher level of artifact compared to those acquired at basic research sites (e.g. due to the limited scan time), leading to different performance of post-processing techniques; and (ii) several established retrospective correction methods can improve data quality most efficiently at tissue boundaries (e.g. ACID robust fitting is most powerful at the interface between WM and CSF (Mohammadi et al., 2013)), while relevant group differences are often located at the tract centers (Grabher et al., 2016).

Although retrospective correction approaches have been shown to significantly improve data quality in the brain, the generally higher noise level in spinal cord DTI might lead to irreversibly corrupted voxels. Such remaining artifacts can bias DTI index maps at single-subject level and introduce an additional variability beside the inherent anatomical variability at group-level. This bias varies with different level of noise and artifacts depending on acquisition-related parameters (sequence, number of diffusion-weighted directions, etc.) and the investigated cohort, which might be one source for the wide range of FA values reported within the healthy spinal cord (mean FA: 0.41–0.85 (Benedetti et al., 2010, Song et al., 2011); standard deviation: 0.02–0.22 (Song et al., 2011, Pessôa et al., 2012)). Importantly, in studies involving multiple groups, increased variability in DTI index maps can make a given effect size between groups more difficult to detect.

In this paper, we introduce a novel outlier rejection technique (*reliability masking*) which is designed to supplement existing correction approaches by identifying and excluding unreliable voxels based on the associated model-fit error of the diffusion tensor. It performs an automatic clean-up of artifactual voxels by comparing the model-fit error to a threshold value. To investigate the effect of the new reliability masking approach as compared to established post-processing methods, we tested how the statistical power of a previously reported clinical finding is affected by (i) registration-based motion and distortion correction, (ii) a chain comprising registration and robust fitting, and (iii) a chain comprising registration, robust fitting, and reliability masking. The clinical finding reported earlier (Grabher et al., 2016) showed decreased fractional anisotropy (FA) in the posterior column and lateral corticospinal tracts above the lesion in patients with cervical spondylotic myelopathy (CSM) when compared to healthy volunteers. Reliability masking and other retrospective correction methods discussed and introduced in the paper are implemented in MATLAB (The MathWorks Inc., Natwick) and will be integrated into the freely available ACID toolbox (www.diffusiontools.com).

## 2 Methods

### 2.1 Subjects

In this study, the DTI data of 21 healthy volunteers (8 female, age: 41.0 ± 11.4 years) and 20 patients with cervical spondylotic myelopathy (CSM) (6 female, age: 52.0 ± 14.5 years) from a previously published study (Grabher et al., 2016) were reanalyzed. The original study was approved by the Ethics Committee of Zurich (ref. number: EK-2012-0343), and all participants provided written informed consent prior to study enrollment. The stenosis (abnormal narrowing of the spinal cord) was at C5/C6 for 13 patients, at C6/C7 for 3 patients, at C3/C4 for 2 patients, and at C4/C5 and C7/C8 for one-one subject, respectively. For more information on patient demographics, see Grabher et al., 2016.

### 2.2 Data acquisition

Scanning was performed on a 3T Skyra MRI scanner (Siemens Healthcare, Erlangen, Germany) equipped with a RF body transmit coil and a standard 16-channel receive-only head and neck coil. To reduce involuntary motion in the neck area, participants wore an MRI-compatible cervical collar (Laerdal Medicals, Stavanger, Norway). First, a 2D T2-weighted turbo spin-echo sequence was applied to obtain an anatomical reference of the cervical spinal cord. Twenty sagittal slices were acquired with the following parameters: slice thickness of 2.5 mm (10% inter-slice gap), matrix size of 384 × 384, field of view (FOV) of 220 × 220 mm^2^, echo time (TE) of 87 ms, repetition time (TR) of 3670 ms, flip angle of 160°, and readout bandwidth of 260 Hz/pixel.

DTI was performed using a reduced-FOV monopolar single-shot spin-echo EPI (ss-EPI) sequence. Thirty diffusion-weighted (DW) volumes (high diffusion-weighting, b = 500 s/mm^2^) were acquired along with 6 T2w (low diffusion-weighting, b = 0 s/mm^2^) volumes. Four repetitions of each DTI dataset were acquired, resulting in 144 volumes for each subject. Each volume consisted of 10 slices centered at the lower edge of the C2 vertebral body and acquired in the axial-oblique plane, perpendicular to the spinal cord. Acquisition parameters were: slice thickness of 5 mm (10% inter-slice gap), FOV of 133 × 30 mm^2^, matrix size of 176 × 40, in-plane resolution of 0.76 × 0.76 mm^2^ and TE of 73 ms. Cardiac gating was used, acquiring data in blocks of two slices per cardiac cycle (concatenation of 5) with an acquisition window of 350 ms and a cardiac trigger delay of 200 ms. The TR (per volume) and the total acquisition time (TA) depended on the participant's heart rate, with nominal values of 3.5 s for TR and 08:20 min for TA, assuming a period of 700 ms for one cardiac cycle. To avoid fold-over artifacts in the phase-encoding direction resulting from the reduced FOV, phase-oversampling of 50% was used and two spatial saturation bands were placed anterior and posterior to the spinal cord (saturation technique is described in Heidemann et al., 2012). Zero-filling interpolation was used to double the matrix size to 352 × 80 and the apparent in-plane resolution to 0.38 × 0.38 mm^2^.

### 2.3 Motion and eddy-current correction

First, all acquired DTI volumes were cropped to an in-plane matrix size of 80 × 80 (from the original 352 × 80) to exclude non-spinal tissue in the readout direction. To correct for spatial misalignments and distortion caused by bulk motion and eddy-currents, the DTI data underwent an iterative affine registration procedure using a modified version of the *spm_coreg* function as implemented in the ACID toolbox. The algorithm uses a multi-target registration approach which accounts for signal and contrast differences between shells by creating separate registration groups for each shell. Then, each volume is registered to its corresponding target image (in our case all DW images to a DW template and all T2w images to a T2w template).

Both volume- (3D) and slice-wise (2D) registration were applied for comparison purposes. The applied methods with the corresponding degrees of freedom are summarized in Table 1. The abbreviations x, y, and z denote the left-right (frequency encoding), anterior-posterior (phase encoding), and head-foot (slice selection) directions, respectively. Allowed degrees of freedom included translation in the x- and y-direction and scaling in the y-direction, as visual assessment of the DTI dataset revealed the most pronounced movements in these directions. Note that translation and scaling in y are mostly caused by the constant and the linear (y-direction) components of the eddy-currents, respectively (Mohammadi et al., 2010). We did not correct for translation in z, because spinal cord anatomy changes only very slowly in the rostral-caudal direction and the application of cervical collar is also expected to reduce involuntary motion in this direction (Yiannakas et al., 2012). Rotation, shearing, and scaling in other direction were not included either, because these were not substantial and were less robust to estimate.

**Table 1:** Details of the applied registration methods for motion and eddy-current correction. Volume-wise registration (i) and slice-wise registration (ii) were applied on the DTI images, allowing translation in the x- and y-direction and scaling in the y-direction. The non-registered dataset (0) was used for comparison purposes. Note that the 30 degrees of freedom for slice-wise registration include 3 parameters for each slice.

Registration method		Translation	Scaling	Number of parameters
None	(0)	–	–	–
Volume-wise	(i)	x, y	y	3
Slice-wise	(ii)	x, y	y	30

### 2.4 Robust tensor fitting

#### 2.4.1 ACID robust fitting

This robust fitting method implemented in the ACID toolbox (referred to as ACID robust fitting) was based on the work of Mohammadi et al. (2013). In short, the linear regression problem of the tensor fitting is solved by minimizing $\rho \left(\varepsilon_{i}\right)={\left(\omega_{i}\varepsilon_{i}\right)}^{2}$, where $\varepsilon_{i}$ represents the model-fit error associated with acquisition i and $\omega_{i}$ represents a weighting function designed to down-weight acquisitions with high model-fit error (Mangin et al., 2002). Similar to Zwiers (2010), the weighting function was factorized into three components: $\omega_{i}=\omega_{1i}\omega_{2i}\omega_{3i}$. The first two factors have the decaying form of $\omega_{1i}=\text{exp}\left(-{\left[\frac{A_{1}\varepsilon_{i}}{C_{1}}\right]}^{2}\right)$ and $\omega_{2i}=\text{exp}\left(-{\left[\frac{A_{2}E_{i}}{C_{2}}\right]}^{2}\right)$, where $E_{i}$ is the model-fit error averaged across the slice and $A_{1}$ and $A_{2}$ are confidence interval parameters. In this study, we used $A_{1}=0.1$, while $A_{2}$ was set to $A_{1}/3$. $C_{1}$ and $C_{2}$ represent the expected spread of non-outlier residuals and are estimated as $C_{1}=1.4826\cdot median\left(|\varepsilon_{i}|\right)$ and $C_{2}=1.4826\cdot median\left(|E_{i}|\right)$ (Rousseeuw and Croux, 1993). Importantly, $C_{1}$ was spatially smoothed in-plane to improve its estimation and the robustness of tensor fitting (Zwiers, 2010). The third factor $\omega_{3i}$ accounts for the distortion of the model-fit error distribution from taking the logarithm of the DW signal intensity.

#### 2.4.2 RESTORE

The RESTORE algorithm used here was implemented in the CAMINO toolbox (Cook et al., 2006). Details about the RESTORE algorithm can be found in Chang et al., 2005.

#### 2.4.3 Weighted ordinary least squares approach

For comparison purposes, tensor fitting was performed using the weighted ordinary least squares (wOLS) approach as well. The wOLS approach used here was implemented in the ACID toolbox and represents robust tensor fitting with parameters $A_{1}$ and $A_{2}$ set to 0.

For each of the three tensor fitting approaches, following voxel-wise DTI indices were calculated: fractional anisotropy (FA), mean diffusivity (MD), axial diffusivity (AD), radial diffusivity (RD), and root mean square of model-fit error (ε).

### 2.5 Reliability masking

The root mean square model-fit error (in the following referred to simply as model-fit error and denoted by ε) represents the remaining difference between the data and the fitted model and thus indicates to what degree the diffusion tensor model explains the diffusion-weighted data in a given voxel. An increase in model-fit error can be caused by (i) low signal-to-noise ratio (SNR) of the dataset, (ii) high amount of corrupted DTI volumes (outliers), and (iii) an inappropriate tensor model to explain the underlying complexity of diffusion (i.e. the single-tensor model does not describe the DW signal adequately).

Robust fitting can substantially reduce the bias introduced by the signal outliers if the amount of outliers is sufficiently low (i.e. the median of the model-fit error is not substantially increased by the outliers). However, it fails to remove the bias if signal outliers appear more frequently (and thereby substantially increase the median model-fit error), or if the SNR of the dataset is low. In our method, we used the model-fit error map to identify voxels irreversibly biased by a high level of outliers or low SNR. A voxel is considered unreliable if the corresponding model-fit error (ε) exceeds a threshold$\;\varepsilon_{thr}$ determined at group-level. By thresholding the model-fit error map, a binary reliability mask $M_{REL}$ is created:

(1) $$M_{REL}\left(r\right)=\left\{\begin{array}{cc} 1 & if\;\varepsilon \left(r\right)<\varepsilon_{thr}\\ 0 & if\;\varepsilon \left(r\right)\geq \varepsilon_{thr} \end{array}\right\}.$$

In a procedure called reliability masking, $M_{REL}$ is applied on the DTI index maps to exclude non-reliable voxels from the analysis.

### 2.6 Spatial normalization

All DTI index maps (including model-fit error maps) were spatially normalized to a self-constructed template that shares the same physical coordinates with the MNI-Poly-AMU template (Fonov et al., 2014). The template was created by (i) registering the individual DTI maps in the control group to the MNI-Poly-AMU template (res.: 0.5 × 0.5 × 0.5 mm^3^) using the *spm_coreg* algorithm and MD and T2w as source and target image, respectively, (ii) averaging all maps, and (iii) reslicing the resulting image to a resolution of 0.2 × 0.2 × 1.0 mm^3^). To use complementary contrast information, the normalization to the so created template was driven by the DTI index maps, rather than by the DW volumes. Non-linear registration of the DTI index maps was performed using the FA voxel-based statistics (FA-VBS) toolbox (Mohammadi et al., 2012c) with refined spatial normalization parameters and taking the anatomy of the spinal cord into account (e.g. the degree of freedom of the spatial transformation along the z-direction was reduced due to the symmetry of the cord in this direction). After normalization, all images were resliced to the native resolution (0.38 × 0.38 × 5.5 mm^3^). Note that during the normalization, the images were cropped along the z-direction, slightly reducing the FOV in this direction. Consequently, after resampling to the native resolution, the number of slices was reduced from 10 to 9, resulting in one missing slice (slice 10).

### 2.7 ROI generation

**Fig. 1:**
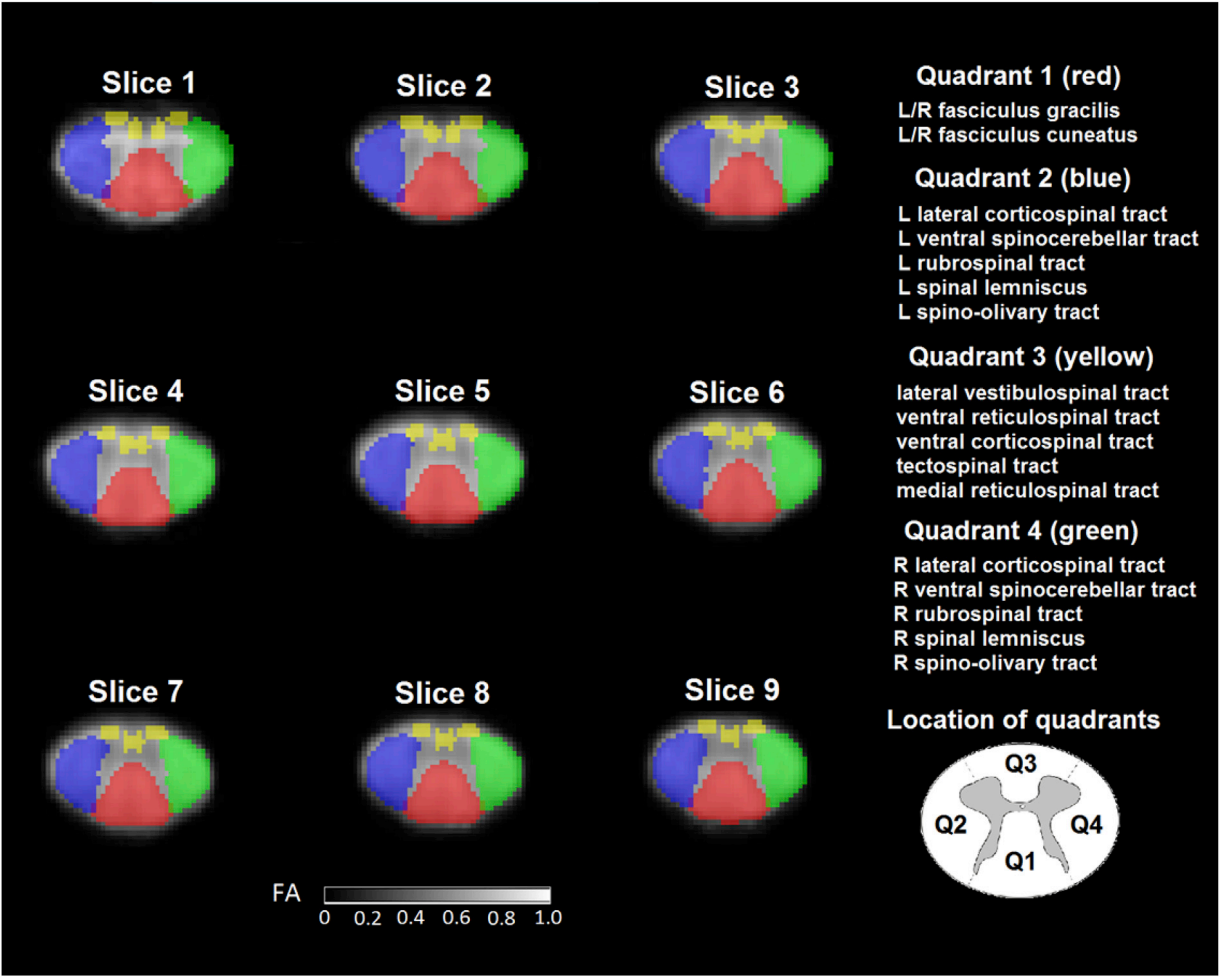
Binary masks of white matter quadrants are illustrated in red (Quadrant 1), blue (Quadrant 2), yellow (Quadrant 3), and green (Quadrant 4) in each slice. The masks are overlaid on the FA template resliced to the native resolution. The schematic locations of the white matter quadrants are also shown at the bottom right. Each quadrant is made up of multiple white matter pathways defined in the Spinal Cord Toolbox and listed on the right. Note that slice 10 is missing, because the images were slightly cropped in the z-direction during normalization, reducing the number of slices from 10 to 9.

Four white matter quadrant masks were created by merging multiple spinal cord pathways defined in the Spinal Cord Toolbox in the form of probability atlases (Levy et al., 2015). The resultant merged probabilistic atlases were thresholded at 0.1 to obtain binary quadrant masks. Care was taken to include only those pathways that did not pose significant risk of partial volume effects with the gray matter (GM). In doing so, only 24 of the total 30 pathways were involved in the quadrant generation. The location and composition of the quadrants are illustrated in Fig. 1. Furthermore, a white matter (WM) mask was also created by merging the four quadrant masks. Quadrant masks were used for the qualitative validation of reliability masking (Fig. 4), while WM mask was used for the rest of the analyses. To account for potential inconsistency between the quadrants and the template and remaining misregistration between the normalized DTI maps and the template, additional subject-specific spinal cord masks were applied on each DTI map. These subject-specific SC masks were drawn manually on the normalized average T2w (b = 0 s/mm^2^) image.

### 2.8 Determining the optimal threshold for reliability masking

To determine the optimal threshold for reliability masking we minimized the standard error of the mean (sem) of the FA sampling distribution in each group. Standard error of the mean measures the precision for an estimated population mean (lower value means higher precision) and is calculated by the formula:

(2) $$\text{sem}\left(FA\right)=\frac{\text{std}\left(FA\right)}{\sqrt{N}},$$

where sem and std denote the standard error of the mean and the standard deviation, respectively, and N denotes the number of voxels (sample size) in the sampling distribution. This approach was based on the idea to remove as many voxels as possible from the heavy lower tail of the FA distribution (as artifacts manifest mostly as lower FA values) but at the same time to remove as few voxels as possible to preserve statistical power.

### 2.9 Statistical analysis

To quantify the effect of reliability masking on the individual DTI maps, both histogram (based on the sampling distribution) and ROI analysis were performed in the WM.

#### 2.9.1 Histogram analysis in WM

All voxels within the intersection of the WM mask and the subject-specific SC masks were pooled across all subjects within a given group (control or CSM group). In this way, two large sampling distributions were created for each DTI scalar map.

#### 2.9.2 ROI analysis in WM

DTI scalar values were averaged within the intersection of the WM mask and the subject-specific spinal cord mask to obtain a single value for each subject.

#### 2.9.3 ROI analysis in SPM cluster

**Fig. 2:**
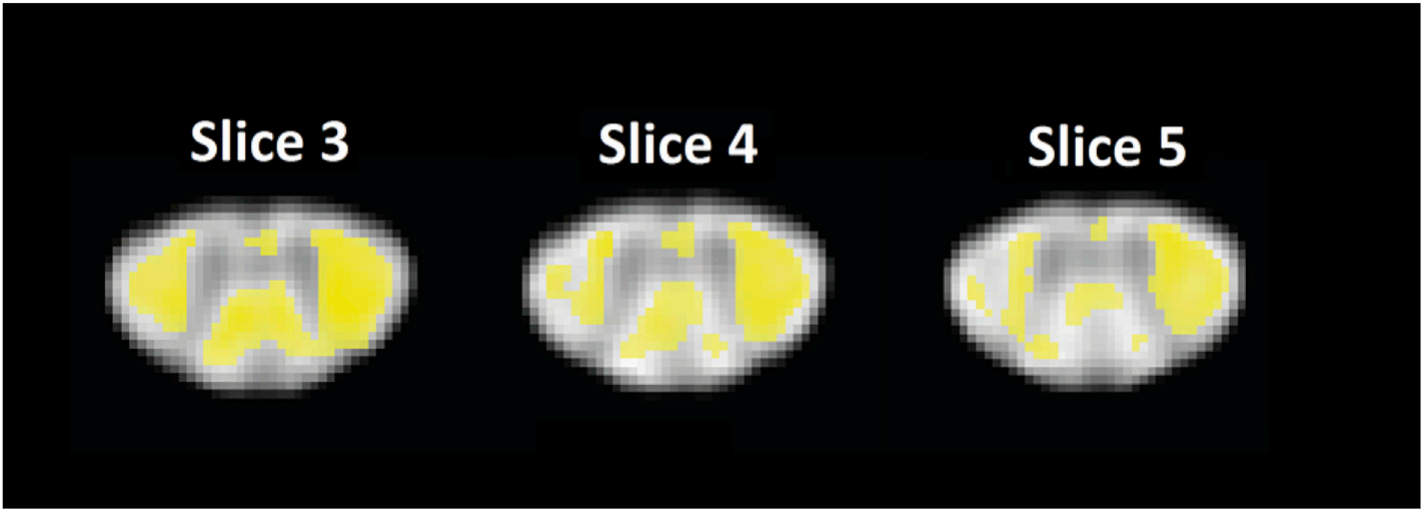
The figure shows a binary mask (yellow) indicating the locations of significant FA differences between CSM patients and controls, overlaid on the FA template. We refer to this binary mask as significant cluster throughout the study and use it to evaluate the effect of artifact correction methods on the investigated clinical finding.

To quantify the effect of post-processing on the investigated FA clinical finding (Grabher et al., 2016), a voxel-wise t-map was created using two-sample *t*-test with unequal variances:

(3) t=mean(FActrl)−mean(FACSM)std(FActrl)2Nctrl+std(FACSM)2NCSM≡''differencebetweenmeans''''standarderrorofdifferencebetweenmeans''

where operators mean and std are performed across subjects within a given voxel. To correct for multiple comparisons, the t-map was thresholded at p = 0.01 (uncorrected) followed by an SPM cluster-level extent threshold of 0.05. Significant clusters were merged into a binary mask representing the areas of FA group differences (Fig. 2). Then, the resulting t-map was averaged within the significant cluster binary mask to obtain a single t-score $\bar{t}$ quantifying the statistical power of the clinical finding. Furthermore, the numerator (“difference between means”) and denominator (“standard error of difference between means”) (sed) of Eq. (3) were also averaged within the significant cluster to obtain single values representing the average voxel-wise difference between means and the average voxel-wise standard error of difference between means, respectively. The procedure was then repeated for each processing chain including (0) no registration + wOLS fitting, (i) registration + wOLS fitting, (ii) registration + robust fitting, and (iii) registration + robust fitting + reliability masking. Note that the voxel removal in reliability masking is taken into account in Eq. (3) in the parameters $N_{ctrl}$ and $N_{CSM}$.

## 3 Results

### 3.1 Determining the optimal threshold for reliability masking (histogram analysis in WM)

**Fig. 3:**
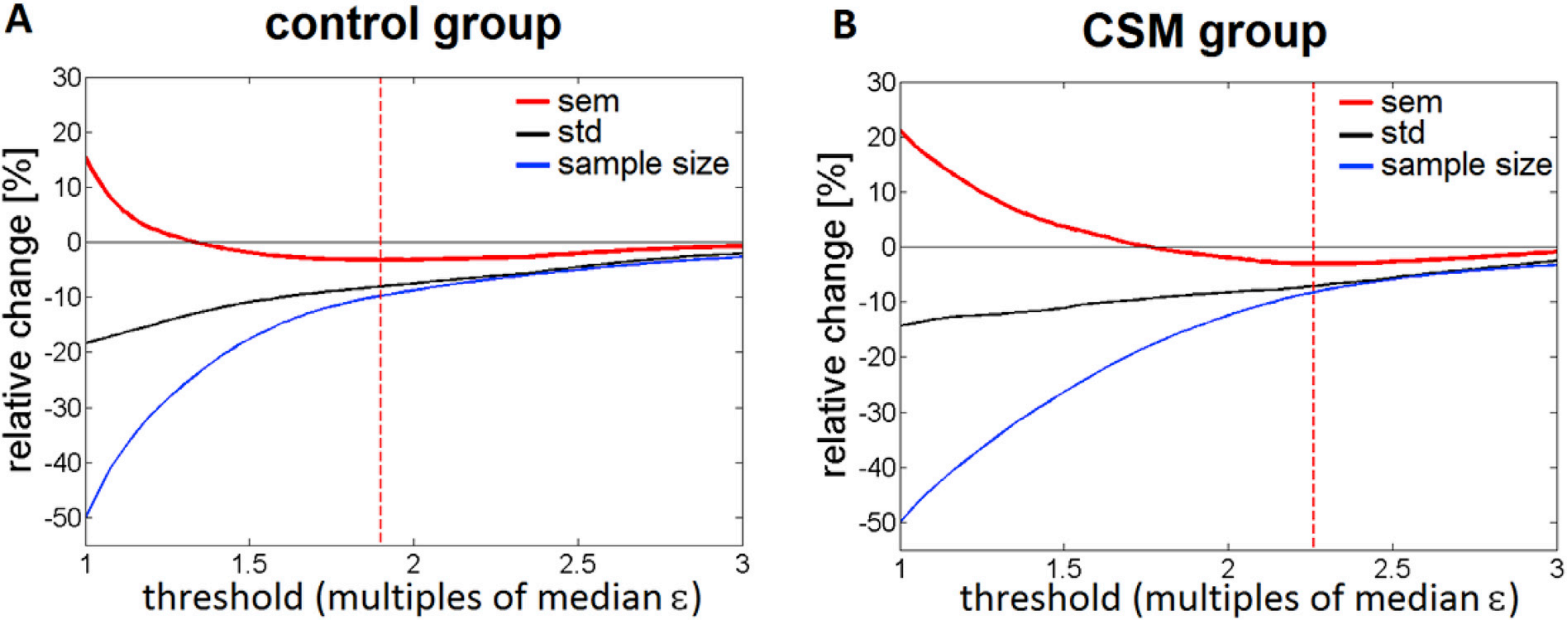
The composite figure shows how reliability masking with varying thresholds ${\text{ε}}_{\text{thr}}$ affects various properties of the FA sampling distribution in the WM including standard deviation, sample size, and standard error of the mean (sem) in the control (A) and CSM group (B). Values are given relative to the data without reliability masking and threshold is expressed in multiples of the median model-fit error ($\bar{\varepsilon }$) in the corresponding group. The threshold resulting in the lowest sem was considered optimal (${\text{ε}}_{\text{thr}}=1.90\cdot {\bar{\text{ε}}}_{\text{ctrl}}$, for the control and ${\text{ε}}_{\text{thr}}=2.26\cdot {\bar{\text{ε}}}_{\text{csm}}$ for the CSM group, indicated by red dashed line).

Both factors composing the standard error of the mean (sem) of the FA sampling distribution in WM (standard deviation and sample size measured via number of voxels N- see also Eq. (2)) decreased continuously with decreasing threshold for reliability masking (Fig. 3). However, their different rate of decrease resulted in a minimum of the sem (red curve in Fig. 3) for both groups, at $1.90\cdot {\bar{\text{ε}}}_{\text{ctrl}}$ for the control and at ${\text{ε}}_{\text{thr}}=2.26\cdot {\bar{\text{ε}}}_{\text{csm}}$ for the CSM group. These threshold values were considered optimal for the corresponding groups and were used in all subsequent analyses.

### 3.2 Qualitative assessment of reliability masking in WM

**Fig. 4:**
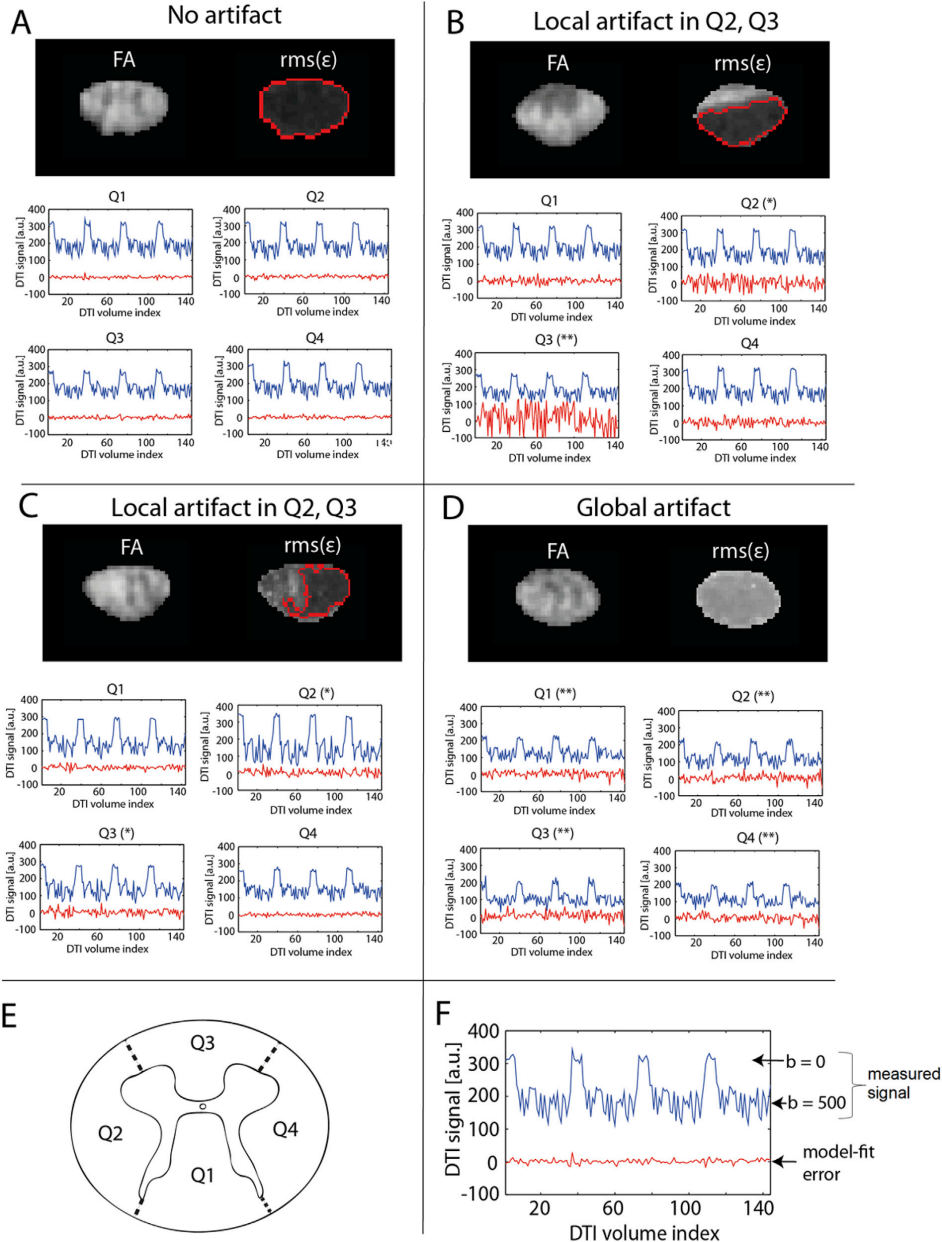
Four examples of how artifacts in spinal cord DTI manifest themselves in the FA map, the map of root-mean-square model-fit error (rms(ε)) (shortly referred to as model-fit error map throughout the paper), and the DTI signal itself: (A) features an artifact-free slice, (B) and (C) show slices with a regional artifact affecting the ventral and left part of the spinal cord, respectively, and (D) shows a more global artifact affecting the whole slice. (E) depicts a schematic spinal cord, illustrating the location of the white matter quadrants (also see Fig. 1). Subplots (A)–(D) are divided into two parts. At the top, FA and model-fit error (ε) maps of the corresponding slice are displayed. At the bottom, the quadrant-averaged DTI signal intensity across all DTI volumes (b = 0 s/mm^2^ and b = 500 s/mm^2^ volumes) in each quadrant is shown (blue line) along with the quadrant-averaged model-fit error (difference between the observation and the model) (red line). Stars above the plots indicate whether the given quadrant is moderately (*) or strongly (**) affected by artifacts. In the model-fit error maps, the red contour lines enclose the areas that are not removed by reliability masking when using the optimal threshold. Note that artefactual voxels in the FA maps are associated with high model-fit error and are effectively removed: in (B) and (C) half of the slice, in (D) the whole slice is removed. Also note that the T2-weighted (b = 0 s/mm^2^) and diffusion-weighted (b = 500 s/mm^2^) volumes are clearly distinguishable in the signal plot with the b = 0 s/mm^2^ volumes (four blocks of six consecutive volumes) having higher intensities than the b = 500 s/mm^2^ volumes (F). In an artifact-free slice (A), the SNR and the contrast between b = 0 s/mm^2^ and b = 500 s/mm^2^ images are high.

Reliability masking was qualitatively validated for its ability to remove voxels contaminated with artifacts with high spatial specificity. Fig. 4 illustrates the performance of reliability masking in different scenarios: in an artifact-free slice (Fig. 4A), in slices affected by isolated local artifacts (Fig. 4B and C), and in a slice with a global artifact (Fig. 4D). If the frequency of outliers among the DW volumes is too high, or in case of low SNR of the dataset, voxels in FA maps will be irreversibly biased and should be excluded from the analysis. Note that there is a great correspondence between artefactual voxels (either clustered in one part of the cord or appearing in the whole slice) and high model-fit error in all of these examples (Fig. 4B–D). Consequently, reliability masking can robustly and automatically remove artefactual voxels with great spatial specificity.

### 3.3 Quantitative assessment of reliability masking (histogram analysis in WM)

**Fig. 5:**
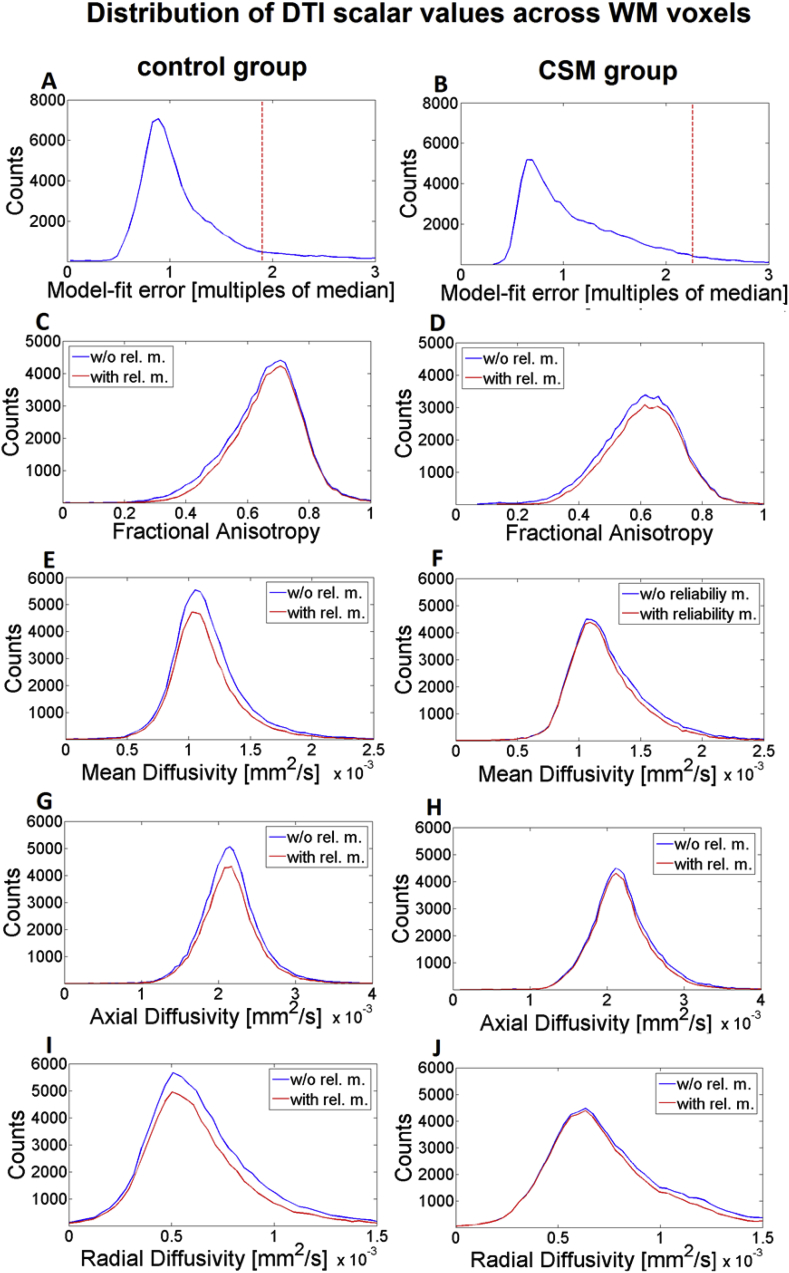
The two upper subfigures show the distribution of the model-fit error in the WM in the control (A) and CSM group (B). The threshold value for reliability masking is indicated by a red dashed line, above which all voxels are excluded during reliability masking. Model-fit error was expressed in multiples of the median value across these voxels ($\bar{\varepsilon }$). The rest of the subfigures (C)–(J) show how reliability masking changes the sampling distribution of DTI indices in both groups. After reliability masking, the distribution of all DTI indices gets narrower and slightly shifted, reducing the standard deviation by 4–10% for all indices and changing the mean by±0–5% (for FA: positive; for MD, AD, RD: negative).

Reliability masking altered the sampling distribution of the DTI indices within the WM, as illustrated for all indices including model-fit error in Fig. 5. The distribution of model-fit error was not Gaussian and was positively skewed toward higher values in both groups (Fig. 5A and B). Reliability masking introduces a cut-off at the threshold value in this distribution (red dashed line in Fig. 5A and B). Notably, reliability masking reduced the negative skewness of the FA distribution and the positive skewness of the MD and RD distributions in both groups, making these distributions more symmetric. The shape of the AD distribution did not change substantially. In accordance with these observations, the standard deviation of all distributions was reduced in both groups, where the highest decrease was found in RD (−9–10%) and the smallest in AD (-4-5%) (Table 2). The reduction in std was slightly higher for the CSM group in all metrics (except for FA). The mean of the distribution was increased for FA and decreased for MD, AD, and MD, although these changes were considerably smaller compared to the standard deviation. Again, the highest and lowest change was found in RD (-3-5%) and AD (−0–1%), respectively, and the changes were higher in the CSM group for all indices.

**Table 2:** Summary of the changes in the sampling distribution of the DTI indices due to reliability masking. The sampling distributions were created by pooling all WM voxels across all subjects in the control and CSM group, respectively.

	FA		MD		AD		RD	
	ctrl	csm	ctrl	csm	ctrl	csm	ctrl	csm
Mean	+1.54%	+1.90%	−1.68%	−2.53%	−0.42%	−1.10%	−3.76%	−4.59%
std	−8.53%	−7.20%	−6.69%	−8.05%	−4.11%	−5.28%	−8.95%	−9.60%

### 3.4 Effect of reliability masking on group-level results (ROI analysis in WM)

**Fig. 6:**
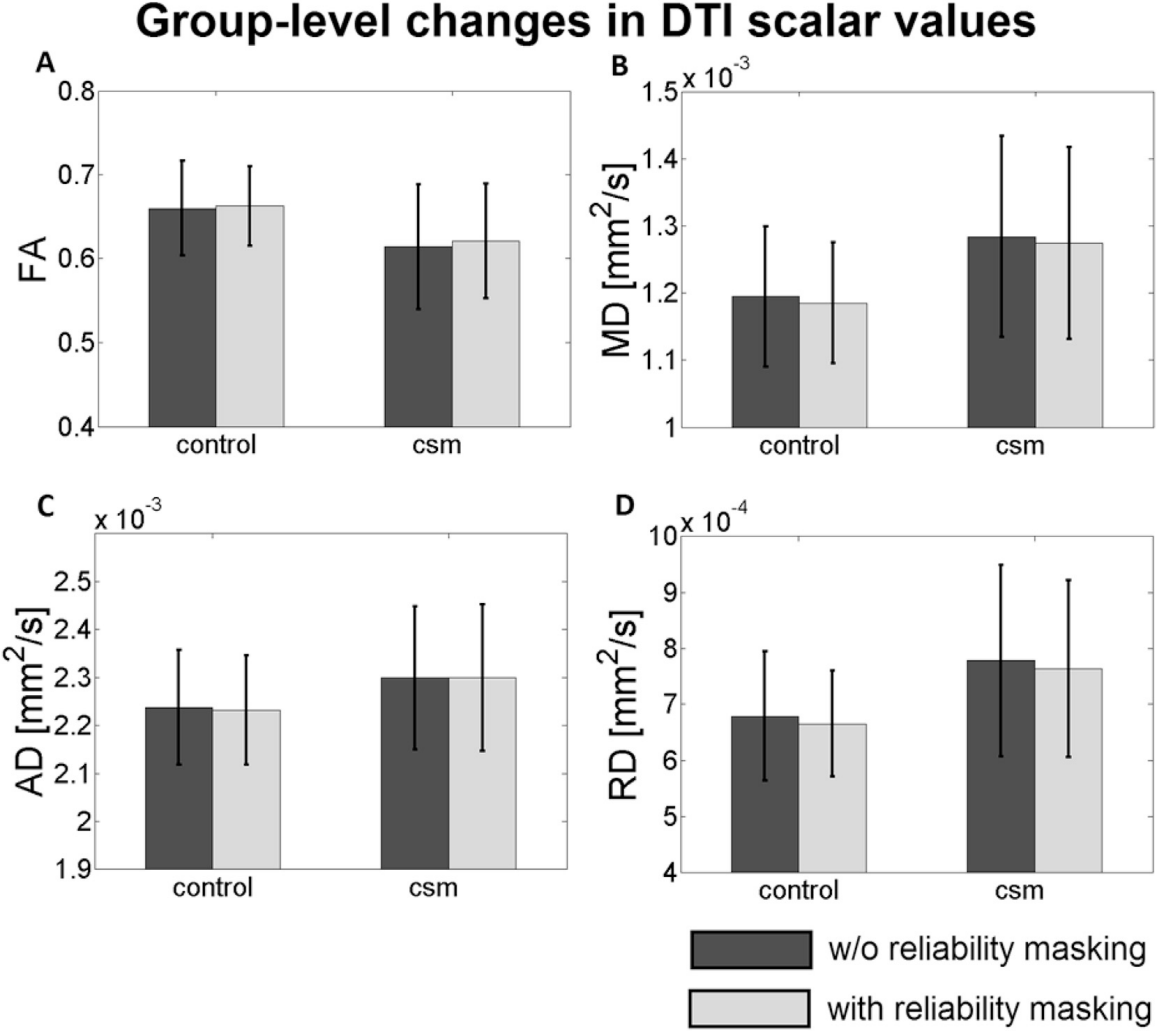
The figure shows the group mean and standard deviation of WM DTI indices before (dark gray bars) and after (light gray bars) reliability masking. DTI maps were generated using ACID robust fitting after slice-wise registration. The group mean of the DTI indices changed only minimally with a slight increase in FA and small decrease in MD and RD. As opposed, the group standard deviation decreased substantially for FA (control vs. CSM group: −15.62% vs. −8.52%), MD (−13.61% vs. −4.59%), and RD (−18.25% vs. −7.58%).

As a consequence of the altered distribution of DTI indices, group-level results were also affected by reliability masking (Fig. 6). While the group mean of the WM DTI indices changed only minimally by ±0–2% (for FA: positive; for MD, RD: negative), its group standard deviation was reduced substantially by 4–18%, with the exception of AD. The highest and lowest changes in mean and std were found in RD and AD, respectively (Table 3).

**Table 3:** Summary of the changes in the group mean and standard deviation of DTI indices due to reliability masking. Group mean and standard deviation were calculated on the DTI indices averaged within the WM (ROI analysis). Note that in contrast to Table 2., data were not pooled across the histogram of all subjects but across the ROI within individual subjects. As a consequence, the sample size corresponds to the number of subjects instead of the number of voxels within the histogram.

	FA		MD		AD		RD	
	ctrl	csm	ctrl	csm	ctrl	csm	ctrl	csm
Mean	+0.45%	+1.09%	−0.81%	−0.73%	−0.22%	+0.02%	−1.98%	−1.83%
std	−15.62%	−8.52%	−13.61%	−4.59%	−4.72%	+2.56%	−18.25%	−7.58%

### 3.5 Comparison of retrospective correction techniques (ROI analysis in SPM cluster)

**Fig. 7:**
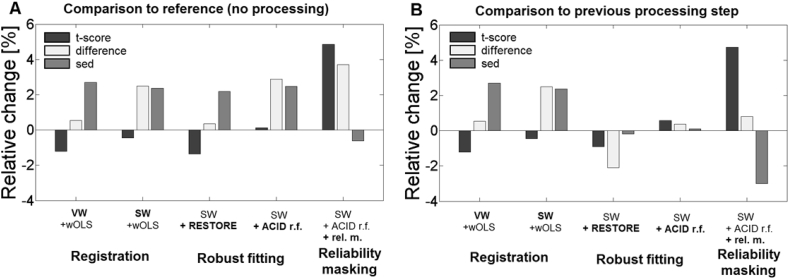
Comparison of retrospective artifact correction methods in terms of their effect on a clinical FA group-difference between controls and CSM patients. Plotted are two-sample t-score (black), difference between group means (light gray), and standard error of the group difference (sed) (gray) averaged within the cluster of significant effect (see Fig. 2) (A). Different chains of artifact correction techniques are tested: 1. registration using volume- (VW) or slice-wise (SW) registration + wOLS fitting, 2. registration + robust fitting using RESTORE or ACID robust fitting, and 3. registration + ACID robust fitting + reliability masking using the optimal thresholds (control: $\varepsilon_{thr}=1.90\cdot {\bar{\varepsilon }}_{ctrl}$, CSM: ${\overset{}{\varepsilon }}_{thr}=2.26\cdot {\bar{\varepsilon }}_{csm}$). The values are given as relative changes compared to the unprocessed dataset. (B) depicts the same processing chains as (A) but the values are given as relative changes compared to the previous chain. SW and VW increased the difference between group means and sed, but overall minimally affected the t-score. Both RESTORE and ACID robust fitting affected the sed minimally, while the difference between groups was decreased by RESTORE. However, their influence on the t-score was rather small. Reliability masking increased the t-score and decreased the sed, leaving the difference between group means almost unaffected.

We tested how different chains of retrospective correction techniques affected the clinical spinal cord DTI finding compared to the unprocessed case (Fig. 7A). Both volume- (VW) and slice-wise (SW) registration (in combination with wOLS fitting) increased the difference between group means (VW: +0.5%, SW: +2.5%) and the standard error of the difference between group means (sed) (VW: +2.7%, SW: +2.4%), overall minimally affecting the t-score (VW: −1.2%, SW: −0.5%). A chain comprising SW registration and either of two different robust fitting methods (RESTORE or ACID robust fitting) produced similar results as the previous chain in terms of difference between group means (RESTORE: +0.4%, ACID r.f.: +2.9%), sed (RESTORE: +2.2%, ACID r.f.: +2.5%), and t-score (RESTORE: −1.35%, ACID r.f.: +0.1%). A chain comprising SW registration, ACID robust fitting, and reliability masking yielded higher difference between group means (+3.7%) and slightly lower sed (−0.6%), increasing the t-score considerably (+4.9%).

To disentangle the individual contribution of each chain element to the above changes, we also tested the effect of each additional step compared to the previous one. Robust fitting affected the t-score (RESTORE: −0.9%, ACID r.f.: +0.6%) and the sed (RESTORE: −0.2%, ACID r.f.: +0.1%) only minimally, while the difference between group means was decreased for RESTORE (RESTORE: −2.1%, ACID r.f.: +0.4%). Application of reliability masking on the SW registration and ACID robust fitting chain had little effect on the difference between group means (+0.8%), but considerably reduced sed (−3.0%) and increased t-score (4.7%).

## 4 Discussion

This paper investigates how established post-processing steps for artifact correction (i.e. registration and robust fitting) and a novel outlier rejection technique (*reliability masking*) introduced in this paper can improve the statistical power of a previously described clinical finding of reduced FA values between healthy subjects and patients with cervical spondylotic myelopathy. We found that the t-score of this clinical finding was minimally affected by applying established post-processing steps, while supplementing the post-processing pipeline by reliability masking improved the t-score considerably. When separately viewing the two factors that underlie the t-score (i.e. differences between FA group means, and standard error of the difference between group means, sed), we found that reliability masking substantially decreased the sed but had only little effect on the difference in mean FA, suggesting that the gain in t-value is driven by reduced variability in both groups.

### 4.1 Reliability masking

Reliability masking is designed to supplement established retrospective artifact correction techniques such as registration and robust tensor fitting by performing a clean-up of irreversibly biased voxels in the DTI index maps (see Fig. 4). Established robust fitting techniques (e.g. RESTORE (Chang et al., 2005), PATCH (Zwiers, 2010), and ACID robust fitting (Mohammadi et al., 2013)) exclude (down-weight) unreliable data points from the model-fit in an iterative manner (i.e. not all data points are used for model fitting). A common feature of these methods is that they operate at the single-subject level (i.e. in each subject independently). In many situations (high level of outliers, low SNR, etc.), however, voxels are irreversibly corrupted and robust tensor fitting methods fail to fully remove the bias introduced by these artifacts. Reliability masking aims to identify the irreversibly corrupted voxels in the DTI index maps by the corresponding root-mean-square model-fit error (shortly referred to as model-fit error). In contrast to established robust fitting techniques, reliability masking is applied after tensor fitting and removes unreliable data points. In each subject, reliability masking compares the map of model-fit error with a threshold value determined at group-level. Voxels with model-fit error exceeding this threshold are considered unreliable and are discarded from the subsequent analysis. Determining the threshold at group-level ensures that the outlier detection is not affected by globally high model-fit errors in single subjects. It is important to stress that reliability masking has to be treated as a supplementary outlier rejection technique, not a competitor to robust fitting.

When applied on the FA maps, reliability masking preferably removes voxels from the heavy lower-tail of the distribution, thereby decreasing the standard deviation (control: −8.5%, CSM: −7.2%) and slightly increasing the mean (control: +1.5%, CSM: +1.9%) of the FA sampling distribution the WM voxels. This is consistent with the hypothesis that outliers mostly manifest themselves as artificially low FA values (Chang et al., 2012). This notion has also been supported by visual inspection of the FA and the corresponding model-fit error maps: in most cases, excluded areas in FA featured visually recognizable artifacts (see Fig. 4B and D for artificially low FA values). When supplementing the processing chain of SW registration and ACID robust fitting by reliability masking, the statistical power of the investigated clinical finding (as measured by the mean two-sample t-score within the cluster of significant region) was increased by 4.7% (Fig. 7B).

The only input reliability masking requires is the threshold for model-fit error ($\varepsilon_{thr}$). This parameter is critical as it determines the threshold above which a voxel is considered artefactual. The choice of $\varepsilon_{thr}$ also affects the number of excluded voxels (Fig. 3), the sampling distribution of DTI indices (Fig. 5), and group-level results (Fig. 6, Fig. 7). To determine the optimal threshold, we minimized the standard error of the mean of the sampling distribution of the metrics of interest (here FA in the WM). The rationale behind minimizing the FA standard error of the mean across a homogenous pool of voxels (such as spinal cord WM) for determining the optimal threshold is that this approach favors reduction in the FA standard deviation (counteracting the artificially high variability in the presents of artifacts) while at the same time penalizing removal of voxels (taking into account the influence of decreased sample size on the statistical power). Although both the FA standard deviation and the number of voxels are a continuously decreasing function of the threshold (Fig. 3), the sem of FA had a distinct peak in both groups representing the optimal threshold. The distribution of DTI indices (e.g. FA) and model-fit error across the region of interest can vary with acquisition protocols and subject groups. For example, in a clinically important scenario, severely impaired tissue in pathology has altered diffusion profile, where the single tensor model may not hold anymore, potentially leading to increased model-fit error. Thus, in pathological subjects the assumptions of reliability masking (high model-fit error is due to outliers or low SNR) might not hold any more, leading to exclusion of the voxels with pathology, which reduces the effect size of the group difference. In our patient cohort, this phenomenon is probably not that pronounced, as our imaging FOV was rostral to the injury site (in the ‘normal appearing white matter’) in 18/20 patients. Nevertheless, we observed that the distribution of model-fit error was skewed toward higher values in the CSM patients compared to controls. Therefore, we recommend to explore the optimal threshold in each group and study separately.

Since the investigated clinical finding involved an FA group difference, in this paper we primarily focused on the effect of reliability masking on FA. However, we also investigated how reliability masking affects the sampling distributions and the group-level results of other DTI scalar values including MD, AD, and RD in both groups (Figs. 5 and 6 and Tables 2 and 3). Of all the metrics (including FA), the distribution of RD showed the greatest changes due to reliability masking, suggesting that RD is most prone to outliers. As opposed, AD barely showed any changes, suggesting that AD is most robust to outliers.

### 4.2 Robust fitting

We found that robust tensor fitting implemented in ACID only minimally affected the statistical power (+0.6%), while the most commonly used RESTORE approach reduced it by 0.9% compared to wOLS fitting. This difference can be caused by differences in the algorithm, but most probably is due to different parameter settings used in both algorithms. An important parameter for robust fitting is the confidence interval parameter ($A_{1}$ in ACID robust fitting, see methods for details) that affects the range in model-fit error within which volumes are not considered outliers. A higher $A_{1}$ excludes more outliers, but can lead to a less stable tensor fit and noise enhancement as the tensor is fit on a smaller set of data. Finding an optimal $A_{1}$ is thus a tradeoff between removing as many outliers as possible to reduce the bias and keeping as many data points as possible to retain SNR. When using $A_{1}=0.3$ (more aggressive outlier rejection) instead of the default $A_{1}=0.1$ used in this study, we obtained similar t-score changes to RESTORE (−1.1%). In summary, the influence of robust fitting on the t-score depended on the algorithm and parameters used, but the overall effect was rather limited. One reason could be that while our clinical finding was located within WM tracts (Fig. 2), ACID robust fitting has been shown to improve data quality mostly at tissue boundaries (Mohammadi et al., 2013).

### 4.3 Motion and eddy-current correction

Registration-based post-processing techniques have been previously demonstrated to reduce motion and eddy-current related distortion artifacts in the DTI data. In our data, we found that registration minimizes the most prominent motion artifact, the displacement of the cord along the phase-encoding direction (data not shown). We also found that slice-wise registration is superior to volume-wise registration in correcting single slices with large displacements. However, both slice- and volume-wise registrations had minimal effect on the statistical power of the investigated between-group difference (−1.2% and −0.5%, respectively). When combined with robust fitting and reliability masking, we applied slice-wise registration due to its superior performance over volume-wise registration. Note that slice-wise registration precludes correction for through-slice motion. However, we do not consider it as a disadvantage, since spinal cord anatomy changes only very slowly in the rostral-caudal direction and the application of cervical collar is also expected to reduce involuntary motion in this direction (Yiannakas et al., 2012).

### 4.4 Methodological considerations

#### 4.4.1 Effect of post-processing on group differences

Similar to neuroscience and clinical studies, we used two-sample *t*-test to investigate the effect of post-processing methods on the group statistics. As shown in Eq. (3), the resulting t-score is affected by both the difference between group means (numerator of the formula) and the standard error of the difference between means (denominator of the formula) which represents the precision for the estimated difference between means. Since both the true population difference and the true (anatomical) variabilities are unknown, interpretation of t-score is not straightforward. However, while outliers in the dataset do not necessarily affect the group difference (if both groups are equally affected), they do increase the standard error of the difference between means. Therefore, we considered reduction in standard error beneficial as a sign of successful outlier removal and we refrained from interpreting changes in the group difference. Our analysis showed that the 4.7% gain in the t-score due to reliability masking was mainly driven by a decrease in standard error (−3.0%) and to a small degree by an increase in group difference (+0.8%). This is not surprising in light of the fact the reliability masking decreased the group standard deviation to a much higher degree than it increased group mean. Investigating the driving force behind changes in t-score also revealed that despite registration methods had minimal influence on the t-score, they increased both group difference (+0.5% for VW, +2.5% for SW) and standard error (+2.7% for VW, +2.4% for SW), canceling each other's effect. Furthermore, the t-score decrease of 3% due to RESTORE is attributed to a decrease in the group difference of the same amount.

#### 4.4.2 Artifacts in spinal cord DTI

Data exploration including visual inspection of DTI volumes along with DTI index and model-fit error maps is essential to recognize artifacts. Robust fitting and reliability masking works on the same principle but automatizes the clean-up procedure. An advantage of reliability masking is its ability to remove artifactual voxels with great spatial specificity, without the need for excluding whole slices. While several types of artifacts can affect the whole slice (bulk motion, eddy-current related distortions, etc.), other artifacts (cardiac pulsation, respiratory motion, CSF flow, incomplete saturation in reduced FOV imaging, etc.) are localized in a well-defined part of the spinal cord (see Fig. 4B–C). Although we used a cardiac-gated DW sequence, the gating may not be equally effective in all subjects. Imperfect saturation of the outer volume in the reduced-FOV sequence can also lead to local ghosts in the spinal cord, which can be corrected by reliability masking. Another important artifact in spinal cord DTI that can be corrected by reliability masking is partial volume effects (e.g. at the CSF white matter boundary). However, there are more specific and efficient methods for partial volume correction (Levy et al., 2015) and for free-water elimination (Pasternak et al., 2009). In order to reduce the influence of partial volume effects between CSF and white matter and to disregard the obvious quality improvement associated with the exclusion of those voxels at the boundary, we excluded boundary voxels by applying subject-specific spinal cord masks on the normalized DTI maps, which were drawn in a rather conservative way.

#### 4.4.3 Beyond-tensor models

In principle, reliability masking is compatible with any model-based diffusion-weighted imaging method (DTI or higher-order models) that provides an appropriate model-fit error. Depending on the model used, reliability masking inherits all the limitations associated with it. Since the model-fit error is used to identify outlier voxels, any situation where the model fails to describe the underlying diffusion signal might bias the outlier detection. For example, model-fit error in DTI is elevated in voxels where the single tensor model is not valid due to complex fiber configuration (crossing fibers, fanning fibers, etc.), possibly resulting in labeling these voxels as outliers. Although such complex fiber structures do exist in the spinal cord, their effect on the DTI signal is rather negligible compared to the brain.

#### 4.4.4 Applying reliability masking in native or group space

Reliability masking can be performed either before or after normalization of the model-fit error maps. In the before-normalization approach, binary masks created by reliability masking are interpolated during normalization, artificially reducing values in the voxels adjacent to the excluded ones. In the after-normalization approach, model-fit error maps are interpolated during normalization, i.e. model-fit error decreases in voxels with originally high values and increases in voxels adjacent to them, which might slightly change the boundary of the binary reliability mask. We recommend using the ‘after-normalization’ approach, since thresholding the model-fit error maps highly mitigates the effect of interpolation.

#### 4.4.5 Reliability masking in group analysis

Reliability masking removes voxels in the calculated DTI index maps, reducing the number of available voxels for the voxel- or ROI-based analysis. At the optimal threshold, 9.8% and 8.3% of all voxels are removed in the control and CSM group, respectively. In a voxel-wise analysis, reliability masking thus leads to varying degrees of freedom in each voxel. This has to be taken into consideration when designing the experiment and interpreting the results. For example, it is not straightforward to use functional neuroimaging software (e.g. SPM in a VBM-style analysis) to visualize group-differences after reliability masking, since statistical methods require the same sample size across voxels (e.g. for performing multiple comparison).

## 5 Conclusion

We have developed a novel outlier rejecting technique (reliability masking) that supplements established artifact correction methods (registration, robust fitting) and tested its impact on the statistical power of a previously reported clinical finding in spinal cord DTI. We found that reliability masking increased the statistical power of this clinical finding more efficiently than established correction methods. Reliability masking is particularly attractive for increasing the statistical power of neuroscience and clinical research studies, as it efficiently reduces group variability of existing data and thus provides a cost-efficient alternative to increasing the group size.
